# The More You Take It, the Better It Works: Six-Month Results of a Nalmefene Phase-IV Trial

**DOI:** 10.3390/jcm8040471

**Published:** 2019-04-06

**Authors:** Pablo Barrio, Carlos Roncero, Lluisa Ortega, Josep Guardia, Lara Yuguero, Antoni Gual

**Affiliations:** 1 Addictive Behaviors Unit, Clinic Hospital, University of Barcelona, Barcelona 08036, Spain; llortega@clinic.cat (L.O.); tgual@clinic.cat (A.G.); 2 Psychiatry Service, University of Salamanca Health Care Complex, Institute of Biomedicine, University of Salamanca, Salamanca 37007, Spain; croncero@saludcastillayleon.es; 3 Addictive Behavior Unit, Psychiatry Department, Hospital de la Santa Creu i Sant Pau Barcelona, Barcelona 08041, Spain; jguardia@santpau.cat; 4 Addictive Behaviors Unit, Germanes Hospitalàries, Sant Boi, Barcelona 08830, Spain; lyuguero.hbmenni@hospitalarias.es

**Keywords:** drinking reduction, nalmefene, phase-IV trial, 6 months, observational

## Abstract

Background: Alcohol use disorders remain a major health problem. Reduced drinking has been increasingly recognized as a valuable alternative to abstinence. Nalmefene has shown in previous, experimental studies to be a useful tool to aid reduced drinking. However, more data from routine practice settings are needed in order to obtain evidence with high external validity. The aim of this study was to conduct a single-arm phase-IV study with alcohol-dependent outpatients starting with nalmefene for the first time. Here, we present the main effectiveness analysis, scheduled at six months. Methods: This was an observational, multisite, single-arm, phase-IV study conducted among adult alcohol-dependent outpatients who received nalmefene for the first time. The study consisted of four visits: Baseline, 1 month, 6 months, and 12 months. At each visit, drinking variables were obtained from the time-line follow-back regarding the previous month. Satisfaction with medication was also assessed from both patients and professionals with the Medication Satisfaction Questionnaire. A repeated measures mixed model was performed for effective analysis regarding drinking outcomes (reduction in total alcohol consumption and the number of heavy drinking days). Regression analyses were performed in order to find predictors of responses to nalmefene. Results: From a total of 110 patients included, 63 reported data at the six-month visit. On average, patients took nalmefene 69% of days during the month previous to the 6-month assessment. Compared to the one month results, the number of heavy drinking days and total alcohol consumption increased. Still, they were significantly lower than baseline values (outcome evolution over time was from 13.5 to 6.8 to 9.4 days/month, and from 169 to 79 to 116 units/month). A total of 23 patients were considered medication responders. The number of days of taking nalmefene was significantly associated in the regression analysis. Satisfaction was globally high for both professionals and patients and, overall, nalmefene was well-tolerated with no serious adverse events reported. Conclusion: The data provided by this phase-IV study suggest that nalmefene is an effective, well-tolerated treatment for alcohol-dependence in real world, clinical settings.

## 1. Introduction

Alcohol remains a first-order global health problem, with 15 million affected people in the EU [1]. Recent publications in the US also warn about an increase in the prevalence of alcohol use disorders during the last decade [2]. The impact it has on both individuals and society is of an enormous dimension, both medically and economically [3,4]. Several strategies have been applied to decrease the burden of the problem, ranging from public health intervention to individualized psychosocial and pharmacological treatment. 

In the arena of pharmacological treatments, one of the latest incorporations has been nalmefene. The lack of real-world data, the need to assess adverse events in clinical populations, and the critiques surrounding the drug since its approval led to the need of a phase-IV trial. A single-arm, observational, phase-IV study of nalmefene, including 110 patients, was started in four different sites in Barcelona, Spain, in 2015.

Baseline and one month results [5] suggested nalmefene was effective in reducing alcohol consumption. They also showed that it was well-tolerated with no serious adverse events, and the satisfaction with the drug of both professionals and patients was high. 

The study, which was designed to last 12 months, consisted of four assessment points in time (baseline, 1, 6 and 12 months). In this paper we report the results of the main effectiveness analysis which was scheduled at 6 months. 

## 2. Methods

A full description of the methods has been described elsewhere [5]. As a summary, this was an observational, multisite, single-arm, phase-IV study, conducted among adult alcohol-dependent outpatients taking nalmefene for the first time. The study consisted of 4 visits: Baseline, 4 weeks, 6 months, and 12 months.

The main outcome variables of the study were:

(1) Reduction in drinking parameters, measured as a change from baseline in heavy drinking days and total alcohol consumption (units in the previous 28 days). The data were derived from the previous month’s timeline follow-back. As the study was conducted in Spain, one drink was considered to contain 10 g of pure ethanol.

(2) Patient and clinician satisfaction, as measured by the Medication Satisfaction Questionnaire (MSQ) [6,7]. Secondary outcomes included changes in drinking risk-level according to the WHO definitions (very high-risk: More than 100 g of alcohol per day in men and more than 60 in women; high-risk: 60–100 g per day in men, 40–60 g per day in women; medium-risk: 40–60 g per day in men, 20–40 g per day in women; low-risk: 1–40 g per day in men, 1–20 g per day in women). Liver enzymes were also analyzed. 

Other collected variables at baseline included previous history of drug use, psychiatric history, family history of drug and alcohol use, and concomitant or changes in psychiatric medication during the study period. At the study visit, the number of days that patients took nalmefene was also recorded. 

Responders to medication were defined in the same manner as in the 1-month publication (a reduction in daily alcohol consumption of at least 70% or downshift of two categories in the drinking risk-level, according to the World Health Organization (WHO), or a shift to low-risk category)

For effectiveness analysis, the repeated measures linear mixed procedure was used for both heavy-drinking days and total alcohol consumption as main outcomes. Age, sex, and number of days taking nalmefene were entered as fixed effects. We also included the presence of any psychiatric or addictive comorbidity as covariates. For each main outcome, regression coefficients (b), *t*-values and *p*-values were calculated. Statistical significance was set at 0.05. Missing data at 6 months for outcome variables was addressed with the conservative approach of baseline observation carried forward (BOCF). Days of medication intake were imputed to 0 for missing values. A descriptive analysis of the Medication Satisfaction Questionnaire (MSQ) was conducted. A satisfaction analysis and a logistic regression analysis for the detection of significant predictors of medication responders were conducted. Included variables were sex, age, number of days taking nalmefene, presence of comorbid drug use, and presence of psychiatric comorbidity. A descriptive analysis of adverse events was also conducted. 

The study protocol, final approved informed consent document, and all supporting information were submitted to and approved by the institutional review boards of all participating centers. All participants provided written informed consent before taking part in study procedures. The study was conducted in accordance with the International Conference on Harmonization and Good Clinical Practice and the principles of the Declaration of Helsinki.

## 3. Results

### 3.1. Sample

From a total of 110 patients included at baseline, 47 were lost to the follow-up at six months, leaving a total of 63 patients reporting drinking outcomes at six months. Of these, 34 patients were still taking nalmefene. Accordingly, drug interruption was reported by 29 patients (eight due to low efficacy, four due to the achievement of the reduction aim, one due to adverse reactions, two due to the change to an abstinence aim, and 14 due to other reasons). No overdoses were reported. Of the 34 patients still on nalmefene, complete abstinence was reported by six subjects. 

In patients still on nalmefene, the mean number of days of patients taking the drug over the previous month was 19.3 (SD = 11.6). More than half of the patients (57%) took it on a daily basis. Basal characteristics of participants can be observed in Table 1.

### 3.2. Efficacy

**Figure 1:**
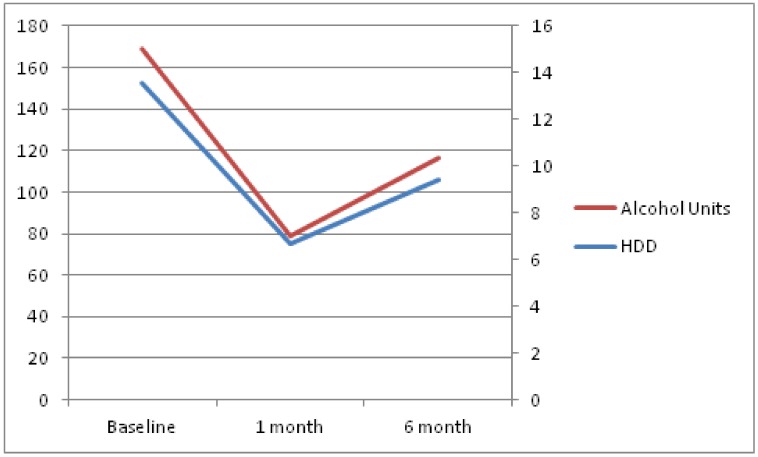
The change over time of study outcomes according to intention-to-treat analysis. The left axis shows alcohol units over the last 4 weeks. The right axis shows heavy-drinking days over the last 4 weeks. HDD: heavy drinking days.

**Figure 2:**
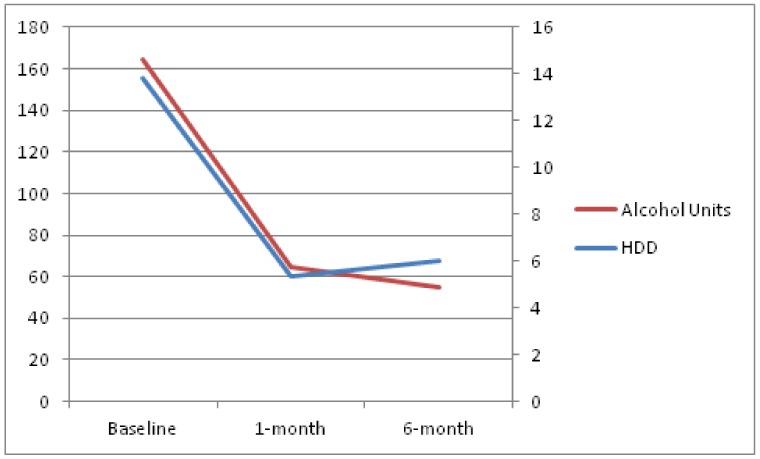
The change over time of study outcomes, according to per protocol analysis. The left axis shows alcohol units over the last 4 weeks. The right axis shows heavy-drinking days over the last 4 weeks. HDD: heavy drinking days.

Both drinking outcomes increased, compared to month 1, but were still significantly reduced when compared to baseline values. The mean number of heavy-drinking days over the last 4 weeks was 9.4 (SD = 10.8) and the total alcohol consumption in units, also over the last 4 weeks, was 116.4 (SD = 171.8). Evolution over time of these parameters can be seen in Figure 1. As a sensitivity analysis, evolution over time, according to per protocol analysis, is provided in Figure 2. Per protocol analysis was conducted using only available data, that is, patients lost to follow-up were not included in this analysis. 

The repeated measures linear mixed model revealed a significant effect in both outcomes at time 1 (first month). Given the increase in both outcomes at time 2 (month 6), no further significant changes were observed. The rest of the covariates were not statistically significant.

A total of 23 patients (21%) were considered medication responders. The only significant predictor found was the number of days with medication intake (OR = 1.058, CI 95% 1.001–1.118). 

### 3.3. Satisfaction

**Figure 3:**
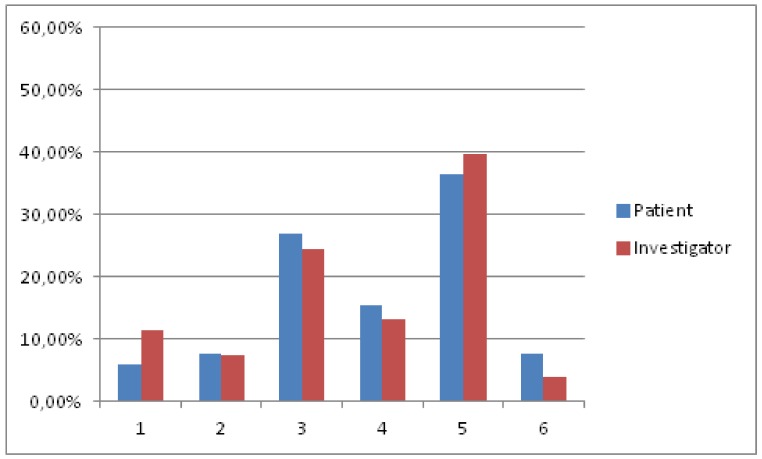
The satisfaction with treatment according to the Medication Satisfaction Questionnaire. 1: Extremely dissatisfied; 2: Very dissatisfied; 3: Somewhat dissatisfied; 4: Neither satisfied nor dissatisfied; 5: Somewhat satisfied; 6: Very satisfied; 7: Extremely satisfied.

Figure 3 displays the satisfaction of professionals and patients with nalmefene at six months, as recorded by the MSQ. In the logistic regression analysis, the the number of days taking study medication revealed a significant effect upon patient satisfaction (OR = 1.074, CI 95% 1.021–1.130).

### 3.4. Safety

At six months, no new drug-related adverse events were notified. That left a total of 29 patients with medication-related adverse events during the first month of treatment. Most events were mild, and no serious adverse events were recorded. Additionally, no overdose was observed or notified. 

## 4. Discussion

The main 6 month effectiveness analysis of this phase-IV trial suggests that nalmefene is effective in reducing alcohol-use when used in real-world, clinical settings. Similar to the 1 month results, nalmefene was well-tolerated and no significant, severe, or life-threatening reactions were observed. There are, however, relevant observations to be made in comparison with previous 1 month results. 

Both heavy-drinking days and total alcohol consumption increased for the whole sample, suggesting nalmefene loses some efficacy over time. It is important to bear in mind that this is, in fact, a common phenomenon to many addiction treatments, whether pharmacological or psychosocial. On the other hand, we took the conservative approach of baseline observation carried forward (BOCF) to deal with missing data, a fact that could have decreased our statistical power. 

It is important to note the relatively small number of patients still taking nalmefene at six months (31% approximately). While low-efficacy and adverse reactions might explain an important share of medication drop-outs, it is also possible that patients who are offered nalmefene are more prone to treatment abandonment, since it has been shown that patients still drinking, and those who aim at reduction objectives, are at greater risk of treatment drop-outs [8,9,10]. 

Taken together, we believe these observations should remind professionals that patients who are prescribed nalmefene are especially prone to abandon treatment, and that efforts should also be directed toward increasing treatment retention. 

Worth mentioning is a similar, recent study [11] conducted among outpatients in routine settings that showed a significant decrease of drinking outcomes at 24 weeks. Similar to our sample, and other previous experimental studies with nalmefene [12], psychiatric comorbidity was high. Interestingly, and contrary to our findings, improvements were seen over the six-month period. All taken together, recent evidence suggests that nalmefene is indeed effective for alcohol use disorder patients in routine settings, where comorbidity is frequent. 

In trying to find differential characteristics between responders and non-responders to treatment, as measured by reductions in alcohol consumption parameters and changes in drinking risk categories, only the number of days taking nalmefene yielded significant effects.

Regarding satisfaction data, a slight decrease in comparison to one month results was observed, probably mimicking the decreased effectiveness at six months. Interestingly, the number of days taking medication was the only covariate associated with increased satisfaction. While it could be interpreted as a consequence, rather than a cause of increased satisfaction, and similar to the regression analysis, which was conducted in order to find predictors of treatment response, we believe this finding suggests that nalmefene might work better over the long-run if taken daily or with a high degree of frequency, rather than sporadically. Another hypothesis worth considering when analyzing this data could be that a higher degree of medication intake is, indeed, a reflection of higher motivation in patients. Therefore, the results obtained in this study are probably not to be entirely attributed to pharmacological effects. It is also fundamental to comment on the fact that we imputed as 0 the days of medication intake in the cases where this variable was missing. While we consider this imputation not unlikely, especially given that nalmefene requires ongoing medical prescription in Spain, it is also true that, in combination with the BOCF imputation for missing drinking outcomes, it could have biased the results obtained, regarding days of medication intake as a significant predictor of both medication response and satisfaction.

Finally, several other limitations apply to this study, such as its observational design and lack of control group, the reduced sample size, and the limited geographical area where the study was conducted. 

## 5. Conclusions

Nalmefene seems to provide further effectiveness at 6 months, in spite of it being reduced, as compared to the first month, after initiating treatment. Our results suggest that a more frequent intake might be related to better outcomes, both in terms of satisfaction and reduced drinking. 

## Figures and Tables

**Table 1:** Baseline sociodemographic and clinical characteristics of included patients.

Characteristic ^a^	Phase-IV (*n* = 110)
Age, years	44.4 (9.4)
Sex male	73 (66.4)
Higher education	30 (27,3)
Organic comorbidity ^b^	30 (27,3)
Age at the onset of drinking problems	23 (12,4)
*Drinking Risk Level*	
Low	50 (45.5)
Medium	18 (16.4)
High	24 (21.8)
Very High	18 (16.3)
g-Glutamyltransferase (IU/L)	84 (128.2)
Alanine aminotransferase (IU/L)	29.2 (15.5)
Aspartate aminotransferase (IU/L)	30,8 (17.6)
Previously treated for alcohol-dependence	51 (46.4)
Previous pharmacological treatment	35 (32,2)
Years of alcohol-dependence untreated	17.5 (12,7)
Previously treated for alcohol-withdrawal	33 (30)
Personal history of psychiatric problems	40 (36.4)
Family history of alcohol problems	53 (48.2)
Addictive comorbidities ^c^ (past or present)	72 (65.5)
Percentage of days taking study medication	70 (64)
Accepts use of alcohol app	52 (47.1)
Monthly heavy drinking days (baseline)	13.5 (11)
Mean alcohol consumption (grams per day; baseline)	60.4 (74,6)

a: Data are expressed as *n* (%) for categorical variables and mean (SD) for continuous variables; b: defined as the presence of diabetes, hypertension, high blood cholesterol or any other significant medical condition; c: defined as any substance use disorder ( except nicotine dependence), past or current, as clinically evaluated in the first visit of the study.

## References

[1] Wittchen H.U., Jacobi F., Rehm J., Gustavsson A., Svensson M., Jönsson B., Olesen J., Allgulander C., Alonso J., Faravelli C. (2011). The size and burden of mental disorders and other disorders of the brain in Europe 2010. Eur. Neuropsychopharmacol..

[2] Grant B.F., Chou S.P., Saha T.D., Pickering R.P., Kerridge B.T., Ruan W.J., Huang B., Jung J., Zhang H., Fan A. (2017). Prevalence of 12-Month Alcohol Use, High-Risk Drinking, and *DSM-IV* Alcohol Use Disorder in the United States, 2001–2002 to 2012–2013. JAMA Psychiatry.

[3] Barrio P., Reynolds J., García-Altés A., Gual A., Anderson P. (2017). Social costs of illegal drugs, alcohol and tobacco in the European Union: A systematic review. Drug. Alcohol Rev..

[4] Miquel L., Rehm J., Shield K.D., Vela E., Bustins M., Segura L., Colom J., Anderson P., Gual A. (2018). Alcohol, tobacco and health care costs: A population-wide cohort study (*n* = 606947 patients) of current drinkers based on medical and administrative health records from Catalonia. Eur. J. Public Health.

[5] Barrio P., Ortega L., Guardia J., Roncero C., Yuguero L., Gual A. (2017). Who Receives Nalmefene and How Does It Work in the Real World? A Single-Arm, Phase IV Study of Nalmefene in Alcohol Dependent Outpatients: Baseline and 1-Month Results. Clin. Drug. Investig..

[6] Kalali A. (1999). Patient Satisfaction with, and Acceptability of, Atypical Antipsychotics. Curr. Med. Res. Opin..

[7] Vernon M.K., Revicki D.A., Awad A.G., Dirani R., Panish J., Canuso C.M., Grinspan A., Mannix S., Kalali A.H. (2010). Psychometric evaluation of the Medication Satisfaction Questionnaire (MSQ) to assess satisfaction with antipsychotic medication among schizophrenia patients. Schizophr. Res..

[8] Haug S., Schaub M.P. (2016). Treatment outcome, treatment retention, and their predictors among clients of five outpatient alcohol treatment centres in Switzerland. BMC Public Health.

[9] Haug S., Eggli P., Schaub M.P. (2016). Drinking Goals and Their Association With Treatment Retention and Treatment Outcomes Among Clients in Outpatient Alcohol Treatment. Subst. Use Misuse.

[10] Meyer A., Wapp M., Strik W., Moggi F. (2014). Association Between Drinking Goal and Alcohol Use One Year After Residential Treatment: A Multicenter Study. J. Addict. Dis..

[11] Di Nicola M., De Filippis S., Martinotti G., De Risio L., Pettorruso M., De Persis S., Giovanni A., Maremmani I., di Giannantonio M., Janiri L. (2017). Nalmefene in Alcohol Use Disorder Subjects with Psychiatric Comorbidity: A Naturalistic Study. Adv. Ther..

[12] Van den Brink W., Sørensen P., Torup L., Mann K., Gual A., SENSE Study Group (2014). Long-term efficacy, tolerability and safety of nalmefene as-needed in patients with alcohol dependence: A 1-year, randomised controlled study. J. Psychopharmacol..

